# Evaluation of individual sensitisation patterns to shrimp allergens in patients with seafood allergy using Immunocap ISAC microarray

**DOI:** 10.1186/2045-7022-4-S2-P41

**Published:** 2014-03-17

**Authors:** Wolfgang Hemmer, Stefan Wöhrl, Gabriele Sesztak-Greinecker, Reinhart Jarisch, Felix Wantke

**Affiliations:** 1 Floridsdorf Allergy Centre, Vienna, Austria

## Background

Seafood is one of the most important causes of anaphylaxis worldwide and considered to be closely associated with house dust mite allergy due to cross-reactivity of mite trypomyosin (Der p 10) with the tropomyosins from shrimps and molluscs. We assessed the prevalence of sensitization to tropomyosin and other shrimp allergens in seafood-allergic subjects from Central Europe and explored the relationship with house dust mite allergy.

## Methods

Sera from 108 patients (43m/65f, mean age 37.7±15.7 yrs) with a convincing history of an adverse reaction to shrimp and/or molluscs and a positive routine test result to any seafood (skin prick test and/or specific IgE) were retrospectively analyzed by ImmunoCAP ISAC® for IgE antibodies against tropomyosin (nPen m 1, rDer p 10, nBla g 7), shrimp arginine kinase (nPen m 2), shrimp sarcoplasmatic calcium-binding protein (nPen m 4), as well as the major dust mite allergens nDer p /f 1 and rDer p/f 2.

## Results

67/108 sera (62%) reacted with at least one shrimp allergen in the microarray, whereas 38% were negative despite most of them showing a positive CAP result to crude shrimp or mollusc extracts. Sensitization to Pen m 1 was most prevalent (42.6%) followed by Pen m 4 (25.0%) and Pen m 2 (13.9%). 73% of the patients were monosensitized to only one molecule, mainly to Pen m 1 (65%) or Pen m 4 (63%) while sensitization to Pen m 2 was rarely monovalent (13%) and characterized by mostly low sIgE concentrations. Only 23/67 sera (34.3%) were positive for the dust mite allergens Der p/f 1 or 2. Correlating sensitization profiles with symptom severity after ingestion of seafood revealed that tropomyosin sensitization is regularly associated with systemic reactions while symptoms are often milder in case of sensitization to Pen m 2 and 4. Reactions were most severe on average in those with negative ISAC.

## Conclusions

Although tropomyosin can be confirmed as an important seafood allergen also in this population from Central Europe, it accounts for only 43% of cases. 38% of seafood-allergic subjects are sensitized to unknown allergens other that Pen m 1, 2 or 4. IgE to Der p/f 1 and 2 is only inconsistently found questioning a close causal relationship between house dust mite sensitization and seafood allergy.

